# Carbapenem-resistant Gram-negative bacteria – analysis of the data obtained through a mandatory reporting system in the Rhine-Main region, Germany, 2012–2015

**DOI:** 10.3205/dgkh000270

**Published:** 2016-04-28

**Authors:** Ursel Heudorf, Barbara Büttner, Anja M. Hauri, Petra Heinmüller, Klaus-Peter Hunfeld, Martin Kaase, Niels Kleinkauf, Sabine Albert-Braun, Rolf Tessmann, Volkhard A. J. Kempf

**Affiliations:** 1 Public Health Department of Frankfurt/Main, Germany; 2 Hessian State Health Office, Dillenburg, Germany; 3 Institute for Laboratory Medicine, Microbiology and Infection Control, Northwest Medical Center, Frankfurt/Main, Germany; 4 National Reference Center for Multidrug-resistant Gram-negative Bacteria, Ruhr University, Bochum, Germany; 5 Institute for Laboratory Medicine, Klinikum Frankfurt Höchst, Frankfurt/Main, Germany; 6 Berufsgenossenschaftliche Unfallklinik, Frankfurt/Main, Germany; 7 Institute for Medical Microbiology and Infection Control, University Hospital Frankfurt, Frankfurt/Main, Germany

**Keywords:** multidrug-resistant Gram-negative bacteria (MRGN), carbapenem-resistant Gram-negative bacteria (CRGN), carbapenemases, mandatory reporting system

## Abstract

**Background:** Multidrug-resistant Gram-negative bacteria (MRGN) and the infections they cause are a serious threat and a challenge to the healthcare system. This particularly applies to carbapenem-resistant Gram-negative bacteria (CRGN). Currently, the introduction of a nationwide mandatory notification system for CRGN in Germany is under consideration. Against this background, this paper presents an analysis of the mandatory reporting system for CRGN in effect since November 2011 in the federal state of Hesse (Germany).

**Materials and methods:** All carbapenem-resistant Gram-negative bacteria and the detected carbapenemases reported to the public health department of the city of Frankfurt am Main, Hesse, Germany, on the basis of the mandatory notification system were analyzed.

**Results:** 827 CRGN cases were reported to the public health department of Frankfurt/Main between April 2012 and December 2015. The following bacterial species were reported: *Pseudomonas* spp. (n=268), *Acinetobacter* spp. (n=183), *Klebsiella* spp. (n=195), *Enterobacter* spp. (n=77), *Escherichia coli *(n=75) and others (n=29). Between 2012 and 2015, a reduction of the CRGN reports was noticed, mainly due to changes in the reporting of *Pseudomonas* spp. Between 2012 and 2015, the total number of notifications decreased slightly, although the number of reported CRGN in screening samples increased, thus giving no indication of a decreased testing frequency. For 10.5% of the patients, the place of residence was not Germany, 18.0% of the patients had previously stayed in hospitals abroad, often in countries with a high CRGN prevalence. CRGN bacteria were reported from all of Frankfurt’s hospitals, and 3.9% were reported from out-patient care facilities. Carbapenemases were detected and reported in 251 CRGN bacteria, including 73 OXA-48, 76 OXA-23, 56 NDM subtypes, and 21 KPC subtypes. There have been no major epidemiological signs of outbreak scenarios.

**Discussion:** CRGN bacteria are already widespread in patients from hospitals and out-patient care facilities. Clearly, infection control measurements should therefore not only include hospital patients but also those receiving out-patient care. Screening strategies focused on patients from foreign countries with high MRGN prevalence is not sufficient, as only 10.5% of MRGN patients resided in those countries, and only 18% of the patients had been previously treated in a foreign hospital. In a public health context, infection control measures should therefore encompass broader screening strategies.

## Zusammenfassung

**Hintergrund:** Multiresistente gramnegative Stäbchenbakterien (MRGN) und dadurch verursachte Infektionen sind eine Bedrohung und große Herausforderung für das Gesundheitswesen. Das betrifft insbesondere Carbapenem-resistente gramnegative Bakterien (CRGN). Derzeit wird überlegt, eine bundesweite Meldepflicht für CRGN in Deutschland einzuführen. Vor diesem Hintergrund wird eine Analyse der seit Ende 2011 in Hessen geltenden Meldepflicht für CRGN vorgelegt.

**Material und Methode: **Alle dem Gesundheitsamt Frankfurt auf der Grundlage der Hessischen Meldepflicht gemeldeten Erreger mit Resistenz gegen Carbapeneme (4MRGN nach KRINKO) und der nachgewiesenen Carbapenemasen werden vorgestellt.

**Ergebnisse:** Von April 2012 bis Dezember 2015 erreichten das Gesundheitsamt 827 Nachweise von CRGN, darunter 268 Nachweise von *Pseudomonas* spp., 183 Nachweise von *Acinetobacter* spp., 195 *Klebsiella* spp., 77 *Enterobacter* spp., 75 *Escherichia coli* und 29 andere. Von 2012 bis 2015 ist insgesamt ein leichter Rückgang der Meldungen erkennbar, bedingt im Wesentlichen durch eine Änderung der Meldepflicht für *P. aeruginosa*. Über die Jahre hat der Anteil der gemeldeten Nachweise aus Screeningproben zugenommen, d.h. es liegt kein Hinweis auf eine abnehmende Untersuchungsfrequenz vor. Fast 10,5% der Patienten hatten ihren Wohnsitz im Ausland, 18% hatten einen vorangegangenen Klinikaufenthalt im Ausland angegeben, häufig in Ländern mit bekannt hohem CRGN-Risiko. CRGN wurden aus allen Frankfurter Kliniken gemeldet, 3,9% auch aus ambulanten Einrichtungen. Bei 251 CRGN-Bakterien wurden Carbapenemasen nachgewiesen und gemeldet, darunter u.a. 73 OXA-48-, 76 OXA-23-, 56 NDM-, 21 KPC-Subtypen. Hinweise auf größere Ausbrüche ergaben sich nicht.

**Diskussion:** Der Nachweis von CRGN bei Patienten in allen Kliniken und auch in ambulanten Praxen zeigt, dass diese Erreger schon weit verbreitet sind. Nicht nur in der Klinik, sondern auch im ambulanten Bereich muss mit dieser Problematik sachgerecht umgegangen werden. Ein Screening allein bei Patienten mit Auslandsanamnese greift zu kurz, da nur bei ca. 10,5% der Patienten mit CRGN der Wohnort im Ausland liegt und nur bei ca. 18% der Patienten ein vorausgehender Aufenthalt in einem ausländischen Krankenhaus stattgefunden hatte. Aus krankenhaushygienischen Gründen müssen deshalb umfassendere Screening-Konzepte entwickelt werden.

## Introduction

Multidrug-resistant pathogens and the infections they cause are a serious threat and challenge to the health care systems of many countries, including Germany [1], [2], [3], [4], [5], [6]. Patients with a history of hospitalization in a country with a high prevalence of antibiotic resistance are considered to be at high risk of colonization or infection with multidrug-resistant Gram-negative bacteria (MRGN) [7], [8], particularly carbapenem-resistant Gram-negative bacteria (CRGN). For example, in January 2015, the cross-border transfer of a German patient from a Turkish hospital to Kiel gave rise to an outbreak caused by a carbapenemase-producing *Acinetobacter baumannii *strain (University Hospital, Kiel, Germany). By February 2015, 13 of 31 patients had died [9].

Given this background, in March 2015, the German Federal Ministry of Health presented a 10-point program to combat antibiotic-resistant pathogens [10]. This revision of the German Antimicrobial Resistance Strategy (DART) [11] has been regarded as an important contribution to the Global Action Plan for the Containment of Antibiotic Resistance by the World Health Organization. Furthermore, the issue of antibiotic resistance was also addressed at the G7 summit in June 2015 at Schloss Elmau, Germany.

While nearly half of the European countries have introduced a national mandatory reporting system for CRGN to the health authorities, Germany currently lacks such a system [6]. Therefore, the 10-point program includes the introduction of mandatory reporting of all laboratory findings of pathogens with carbapenem resistance [10]. The German Federal States, however, have the option to mandate reporting of specified conditions by local state law. The Federal State of Hesse was the first state to introduce a mandatory reporting system for CRGN in late 2011. The experience gathered from Frankfurt and the Rhine-Main area, where legislatively mandatory reporting of CRGN has been in effect for 4 years, is presented [12], [13]. 

## Material and methods

On November 29, 2011, the legal obligation for laboratories to report carbapenem-resistant pathogens went into effect in Hesse, one of the federal states of Germany [12]. According to an implementing decree from April 2012, which specified the reporting criteria, the detection of any carbapenemase and/or carbapenem-resistant Enterobacteriaceae, *Pseudomonas* spp. and *Acinetobacter* spp. from any patient material was to be reported within 24 hours directly to the responsible local health authority. With the publication of the recommendations of the Commission for Hospital Hygiene and Infectious Disease Prevention (KRINKO) on the management and control of MRGN, the criteria for reporting were adapted to the definitions of the KRINKO in April 2013 [7], [8]. The classification of the pathogens is based on the susceptibility of individual pathogens to the most important groups of antibiotics for initial therapy of severe infections (acylureidopenicillins, 3rd and 4th generation cephalosporins, carbapenems and fluoroquinolones). While upholding compulsory reporting of the detection of any carbapenemase, Enterobacteriacea, and *Acinetobacter* spp. resistant to the four clinically most relevant antibiotic classes mentioned above (classified as 4MRGN) from any sampling site (similar to the previous definition), the mandatory reporting of carbapenem-resistant *Pseudomonas aeruginosa* was limited to its isolation from blood and cerebrospinal fluid (CSF) [14], in which case the first detection during every in-patient stay is to be reported. Every notification is counted as a single case. Every detection of a previously unidentified CRGN from a single patient as well as the repeated detection of the same pathogen during multiple hospital stays is counted as a new case. Therefore, multiple cases may be associated with a single patient.

Below, all reports of pathogens with resistance to carbapenems (imipenem, meropenem) received by the public health authority of Frankfurt from April 2013 (the introduction of specified reporting criteria) through December 2015 are presented. These laboratory reports include all notifications from healthcare facilities in Frankfurt/Main, encompassing results from patients who were in Frankfurt (hospital or medical practice) during microbiological sampling, independent of their main or current place of residence, as well as the results of residents of Frankfurt who were treated outside of the city of Frankfurt/Main but whose main place of residence was Frankfurt. Frankfurt/Main is a city in the center of a large metropolitan area (Rhine-Main area), where highly specialized health care facilities are concentrated. By number, 10% of the hospitals in the Federal State of Hesse are located in Frankfurt (17/172), but these include a number of large tertiary care hospitals whose catchment area reaches far beyond the city’s boundaries. The city’s 700,000 inhabitants make up 12% of the state population (6.1 million).

After the notification by the laboratory, the treating physicians were given a questionnaire by the public health authority asking for further data concerning the current hospital stay (date of hospitalization, sampling date, isolation measures, whether the case is part of an outbreak), information on travel history within the last 12 months, and previous hospitalizations in Germany and abroad within the last 6 months.

## Results

**Figure 1:**
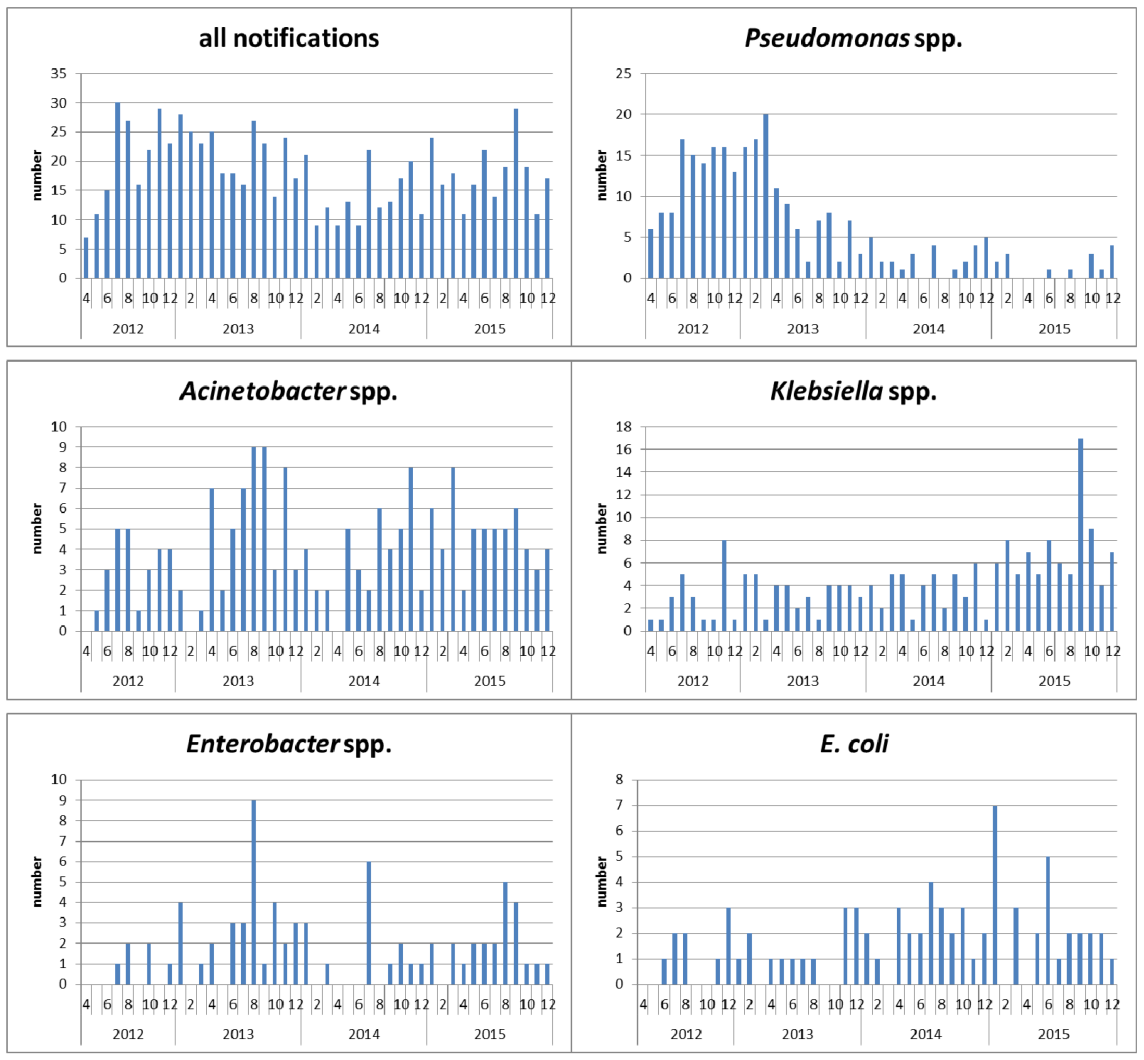
Diagram of the pathogens with acquired carbapenem resistance reported to the public health authority of Frankfurt according to the Hessian mandatory reporting legislation, by month of notification

Between April 2012 and December 2015, the public health authority of Frankfurt/Main received 827 laboratory reports of CRGN. These included 183 detections of *Acinetobacter* spp. (182 *A. baumannii*, 1 *A. calcoaceticus*), 195 *Klebsiella* spp. (192 *K. pneumoniae*, 3 *K. oxytoca*), 77 *Enterobacter* spp. (47 *E. cloacae*, 28 *E. aerogenes*, 1 *E. agglomerans*, 1 *E. sakazakii*), 75* E. coli*, 29 others (11 *C. freundii*, 1 *C. braakii*, 8 *P. mirabilis*, 2 *P. vulgaris*, 4 *S. marcescens*, 3* M. morganii*). The distribution by year is shown in Table 1 (Tab. 1). Figure 1 (Fig. 1) shows the reports of pathogens per month. In 2012, only 9 instead of 12 months were evaluated, taking into account a 3-month start-up phase. From 2013 to 2014, a marked decrease in the reports of *Pseudomonas* spp. was observed, due to a change in the reporting criteria. Up to 2015, a sharp increase in *Klebsiella* spp. is evident. 

CRGN bacteria were reported not only from all of Frankfurt’s hospitals, but also from out-patient settings in Frankfurt and from healthcare facilities outside of Frankfurt for patients whose place of residence was Frankfurt. In total, 89.3% of the reports were from facilities within Frankfurt and 10.8% from facilities outside Frankfurt. Up to 2014, the proportion of reports from out-patient settings increased from 2.2% to 7.1%, with a decrease to 3.7% in 2015 (Table 2 (Tab. 2)).

The permanent place of residence of two-thirds of the patients (n=107; 64.8%) was Frankfurt/Main, a quarter lived outside Frankfurt in Germany, most of whom resided in the surrounding Rhine-Main area, and the permanent place of residence of 10.5% was abroad. Between 2012 and 2015, the proportion of patients from abroad increased from 8.9% to 13.8% (Table 2 (Tab. 2)).

Hospitalization within the 6 months preceding the detection of CRGN was documented for 61.0% of the patients. 18.0% had been hospitalized abroad – with an increasing tendency from 2012 (11.5%) to 2015 (24.9%) (Table 2 (Tab. 2)).

In 173 (20.9%) of the cases, the isolated pathogen was reported as the causative agent of an infection, with a decreasing tendency from 54 (30%) in 2012 to 30 (13.8%) in 2015. In parallel, the proportion of reported cases from nose/throat/skin or rectal swabs, i.e., from screening samples, increased significantly from 2012 (17.8%) to 2015 (53.9%) (Table 2 (Tab. 2)).

On average, Frankfurt’s hospitals reported 10–12 cases/year, with a wide range of 0–87 cases per hospital. 

Regarding the different pathogens, patients with carbapenem-resistant *A. baumannii* and *Klebsiella* spp. are significantly more frequently residents of foreign countries (20.2% and 13.8%, respectively) and/or have been significantly more frequently hospitalized abroad (35.0% and 28.7%, resp.) within the 6 months before the current detection than patients with other carbapenem-resistant pathogens (Table 3 (Tab. 3)). Patients from whom *Enterobacter* spp. and *Pseudomonas* spp. were isolated rarely reported being residents of foreign countries (less than 4%), and only 7.8% of the patients with *Enterobacter* spp. and 4.9% of the patients with *Pseudomonas* spp. reported having been hospitalized abroad. Further details on the respective foreign countries of residence or hospitalization are given in Table 3 (Tab. 3).

In 251 (30.3%) cases, the public health authority received additional information on the detection of a carbapenemase. The most frequently detected carbapenemases were oxacillinases (OXA) (n=155, including 76 OXA-23 and OXA 23-like carbapenemases from *A. baumannii*, 73 OXA-48 from Enterobacteriaceae), followed by 56 New Dehli metallo-β-lactamases (NDM), 21 *Klebsiella pneumoniae* carbapenemases (KPC), and 24 Verona integron-encoded metallo-β-lactamases (VIM). Thirteen pathogens carried two carbapenemases (Table 4 (Tab. 4)).

Between 2012 and 2015, the number of CRGN increased (2012: n=29; 2013 n=68; 2014 n=74; 2015 n=80). The proportion of positive carbapenemase identifications varied markedly for different pathogen species. 

Patients harboring pathogens with a detected carbapenemase more frequently reported a previous hospitalization abroad than did patients with a CRGN without a detected carbapenemase (56% vs. 8%); their place of residence was also more frequently abroad (53% with a detected carbapenemase vs. 3% without a detected carbapenemase).

## Discussion

As early as 2011, the European Health Agency (ECDC) recommended making CRGN a reportable entity [15], [16]. This recommendation was implemented in Hesse in late 2011. Besides the detection of carbapenemase, the legal reporting obligation currently includes all 4MRGN Enterobacteriaceae and bacteria of the *A. baumannii* complex detected from any sampling site, while 4MRGN *Pseudomonas* spp. are only reportable when isolated from blood or CSF.

After the 2013 revison of the notification criteria, which includes a restriction of the mandatory reporting for *Pseudomonas* spp. to samples from blood and cerebrospinal fluid, a drop in the total number of reports received was observed. Since at the same time the proportion of screening tests (nose/throat/skin and rectal swabs) increased from 18% in 2012 to 54% in 2015, it must be assumed that this is not due to a decrease in the number of tests performed.

The proportion of reports classified as CRGN infections (vs. colonization) by the treating physician decreased from 30% in 2012 to 13.8% in 2015. Larger clusters or outbreaks were not observed during the study period, probably as consequence of intensive screening, hygiene, and isolation measures performed by the hospitals. Smaller clusters with detections of *A. baumannii* (colonizations, no notifiable outbreaks with infections) were reported from two hospitals. Comprehensive training had been performed in all hospitals after several outbreaks of carbapenem-resistant *A. baumannii* occurred in 2006 and 2007 in Frankfurt [17], [18], [19], [20]. This training has helped to establish relatively strict hygiene and screening measures in Frankfurt relative to the detection of *A. baumannii*, and these measures have apparently prevented larger outbreaks since. Larger outbreaks due to other CRGN bacteria were not observed. A third of all samples was taken within the first three days after hospitalization. These pathogens must therefore be regarded as acquired outside of the hospital.

Hospitals often compete for elective, well-paying customers from abroad (also from countries with high CRGN prevalence). If these facilities do not take the CRGN risk into account in advance, this can have severe consequences for infection control measures. Large hospitals with in-house laboratories, intensive screening regimes, and good hygiene management may be able to cope with the problem. In hospitals without their own on-site hygiene officer or without sufficient laboratory capacities, admitting such “medical tourists” without a detailed prevention concept may increase the risk of outbreaks and considerably facilitate the unrecognized spread of CRGN.

CRGN cases have been reported from all of Frankfurt’s hospitals as well as from some out-patient facilities since the introduction of the reporting obligation. The hospitals reported 10 cases/year on average, with large differences between the individual facilities (see above). These differences can be caused by a different case-mix in the institutions and by performing screening measures with different degrees of strictness. The proportion of CRGN reports from out-patient settings in Frankfurt was 3.9%. Therefore, the occurrence of these pathogens is no longer limited to large tertiary care hospitals; rather, they are apparently detectable in the community outside of medical facilities.

The KRINKO [7], [8] recommends screening patients with a risk of being colonized or infected with 4MRGN and to preemptively isolate these patients. Risk patients are defined as patients with recent contact to healthcare facilities in countries or regions where MRGN are endemic, and patients who have had contact to other 4MRGN-positive patients.

Looking at the carbapenem-resistance cases reported to the public health authority between 2012 and 2015, it appears that about 60% of the patients were hospitalized in the previous 6 months. 18% of the patients were hospitalized in a foreign country, with an increasing proportion over the years (2012: 11.5%, 2015: 24.9%). Most of the prior hospitalizations took place in countries with known high CRGN prevalences.

Many descriptions of imported CRGN due to transmission by patients with a history of hospitalization abroad support the recommendation to perform a MRGN screening of such patients on admission to a hospital, and to isolate them preemptively until the receipt of the laboratory results. Such an approach may have reduced the extent of the *A. baumannii* outbreak in early 2015 in Kiel, Germany.

On the other hand, it must be mentioned that most of the CRGN bacteria were detected in patients who neither resided nor were hospitalized abroad. Apparently, further risk factors exist. If the screenings were limited exclusively to patients who have a history of travel or residence abroad, many cases could not be detected in advance, but would only be found when diagnosing a manifest infection, with the consequence that hygiene measures would be initiated much too late.

For 251 (30.3%) pathogens, the public health authority received additional information on the detection of carbapenemases. Thirteen pathogens carried two different carbapenemases. Overall, a wide spectrum of carbapenemases was detectable from several different pathogens. OXA-23-like carbapenemases were the most frequently detected type (n=76, only in *Acinetobacter* spp.), followed by OXA-48 carbapenemases (n=73, found frequently in *Klebsiella* spp.) and different NDM carbapenemases (n=56; all from Enterobacteriaceae and *Acinetobacter* spp.). KPC carbapenemases were reported 21 times, with 18 of these from *Klebsiella* spp. Further OXA carbapenemases were almost exclusively found in *A. baumannii* strains. The results of the first year [21] were therefore largely confirmed. Interestingly, the CRGN spectrum does not substantially differ from the 2013 results [22] of the national reference center for Gram-negative pathogens, but can be clearly distinguished from data from other regions in Hesse [23], [24], [25]. These data demonstrate regional differences in the occurrence of cabapenemases in CRGN, the reasons for which are not yet fully understood.

Sixteen to 22% of the patients with reported KPC-, NDM-, OXA-23- and OXA-48-carbapenemase carrying bacteria had their primary place of residence abroad. 20% of the patients with an NDM-, 27% with an OXA-48-, 33% with an OXA-23- and 44% with a KPC-producing pathogen reported a previous hospitalization abroad. However, none of the patients with a carbapenemase-producing pathogen with an OXA-40 and -58 or a VIM carbapenemase reported having been abroad.

A decolonization of CRGN patients currently does not appear feasible. Many CRGN species are detectable over months or even years. In some cases, the (re-)identification succeeded after an interval of several months in which the pathogen was not detectable. The recurrence of CRGN is possibly triggered by an intermittent antibiotic therapy selecting very small populations of bacteria in the patient. This demonstrates the significance of an appropriate administration of antibiotics, and particularly antibiotics exerting a high selective pressure should be prescribed cautiously. Furthermore, in hospitals, timely screening and intensified hygiene and isolation measures are required, in order to stop the further spread of CRGN. This is the reason why reported data should not be evaluated according to the now-common residence principle, but rather by the current location, i.e., with regard to the hospital in which the pathogen was detected [26]. The CRGN burden in the medical institutions in Frankfurt/Main would have been underestimated by half, if the focus had only been placed on the patients with a permanent residence in Frankfurt.

CRGN pathogens are still rarely detected in Germany. The measures planned now by the Federal Ministry of Health, including mandatory reporting of CRGN, in combination with intensified hygiene and isolation measures [10] and cautious administration of antibiotics with high selective pressure may help to counteract the increase of CRGN.

## Conclusion

CRGN pathogens have been detected in all hospitals; they are not limited to tertiary care hospitals. Additionally, because they are increasingly reported from out-patient settings, appropriate management must also be established in such facilities. The detection of many different carbapenemases and many different CRGN species argues against the occurrence of a single, large outbreak.

Only 10.5% of the patients with CRGN have their main place of residence abroad and 18% of the patients have reported a previous hospitalization abroad. This emphasizes that only screening patients with a history of traveling to or living in a foreign country does not suffice. More comprehensive screening concepts must be developed. Apparently, new CRGN bacteria are continuously carried into Germany through travel and medical tourism, the spread of which is favored by inappropriate antibiotic therapy.

## Notes

### Competing interests

The authors declare that they have no competing interests.

### Acknowledgement

The work of Volkhard Kempf on the topic of “Acinetobacter” is supported by the German Research Foundation (DFG FOR 2251).

## Figures and Tables

**Table 1:**
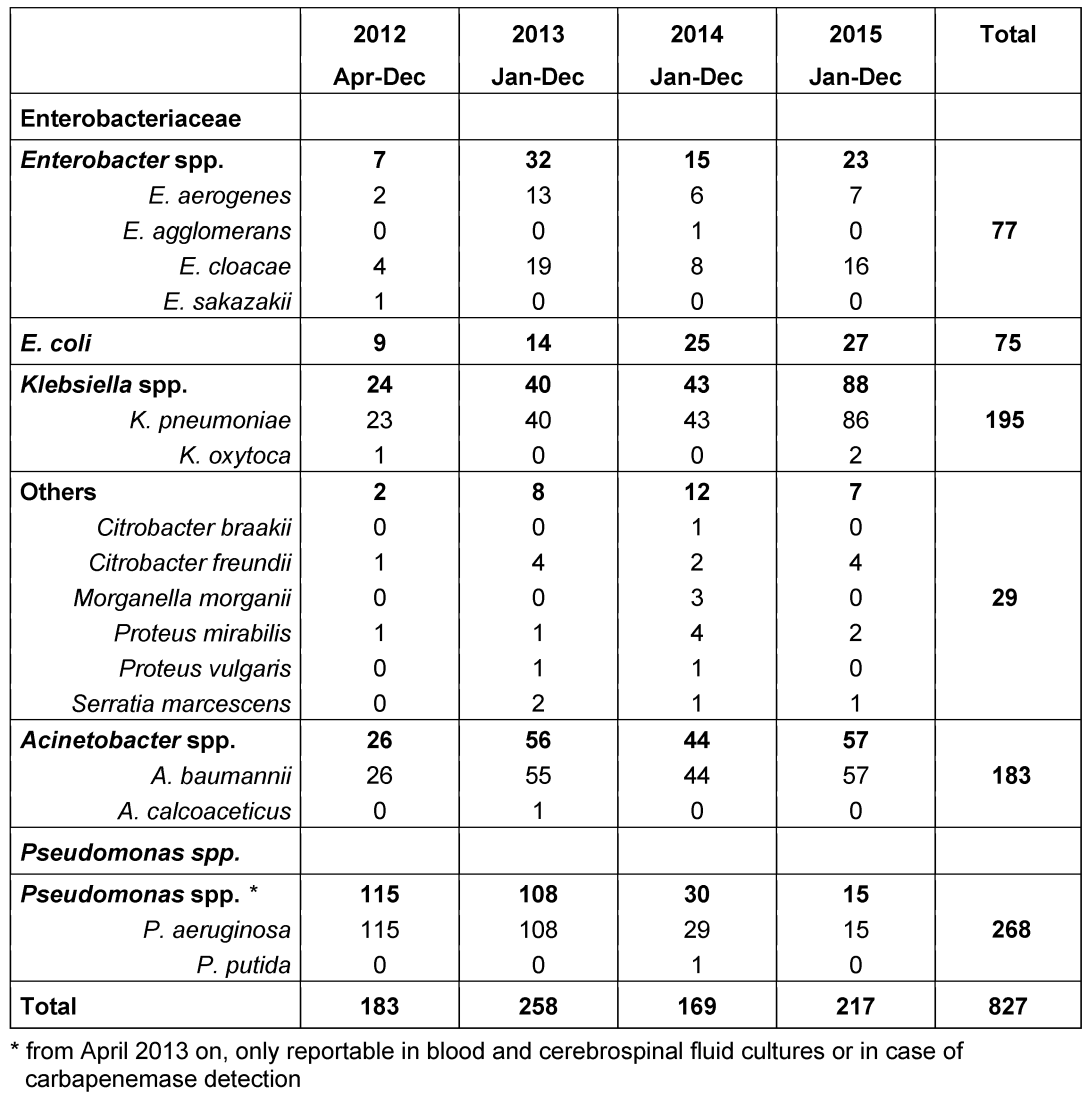
Overview of pathogens with acquired carbapenem resistance reported to the public health authority of Frankfurt according to the Hessian mandatory reporting legislation (April 2012 to December 2015)

**Table 2:**
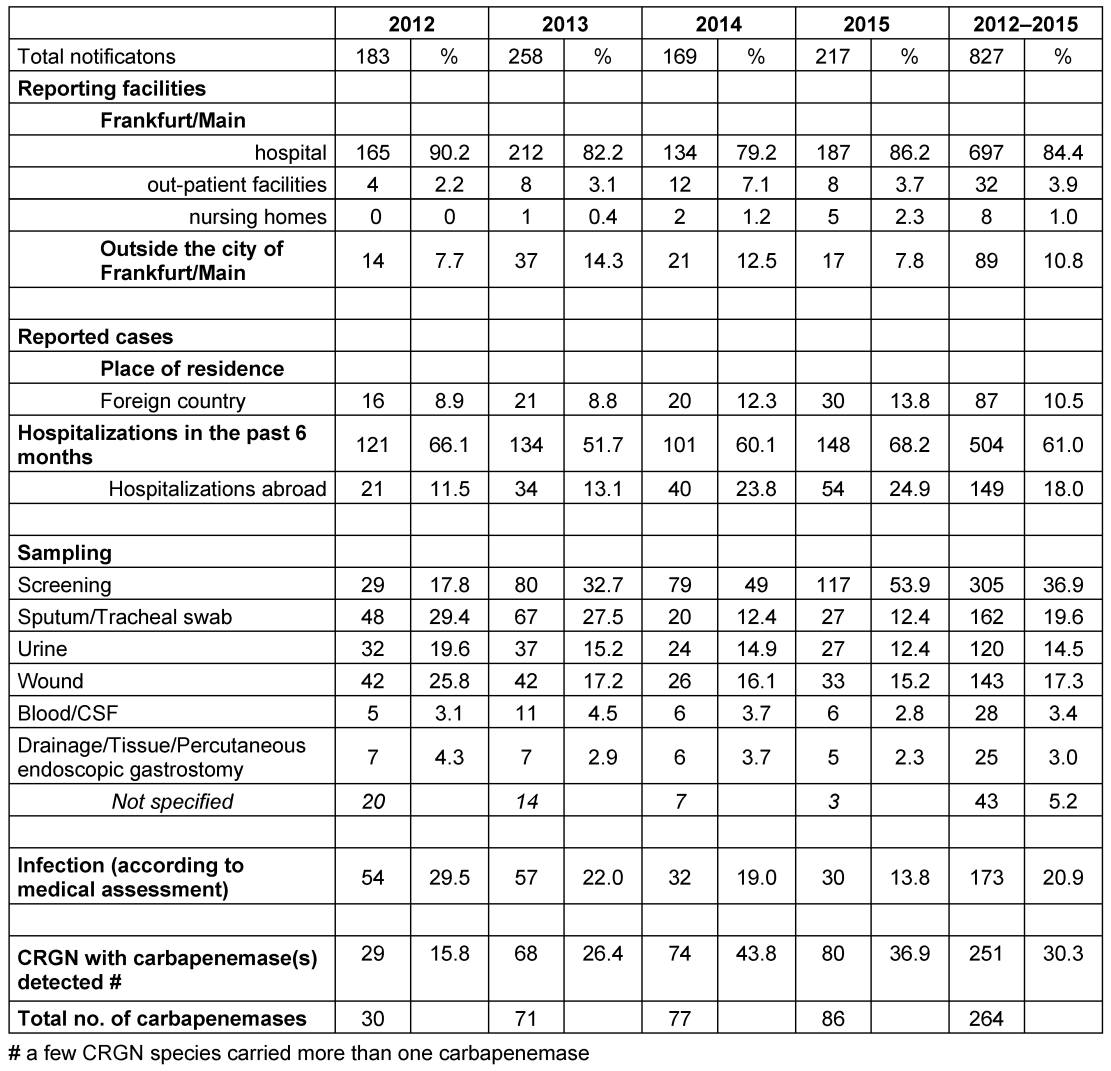
CRGN cases reported to the public health authority Frankfurt between 2012 and 2015 by notifying facility, patient residence abroad, clinical information and patient history, infection and sample type

**Table 3:**
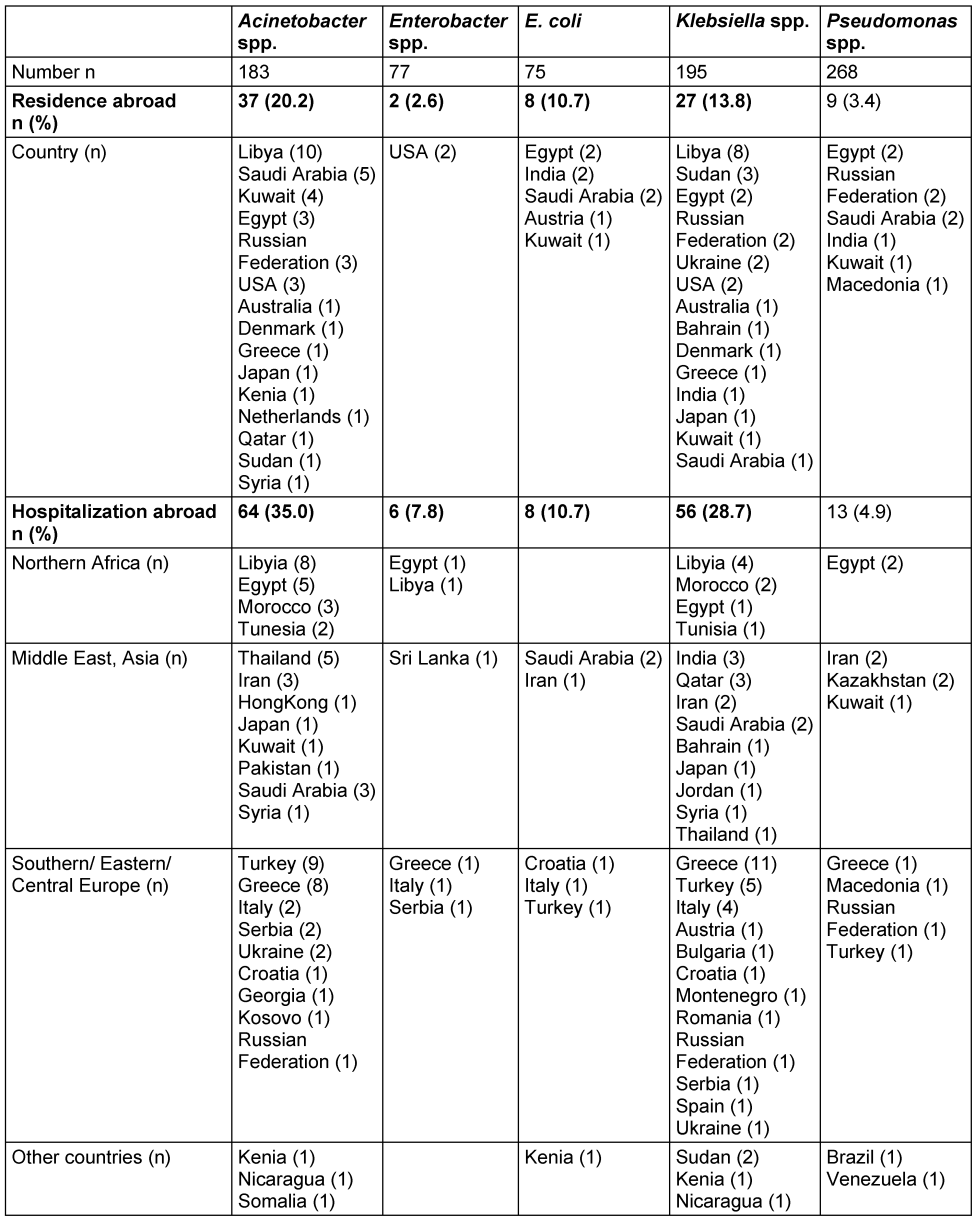
Details on residence and hospitalizations abroad of patients with CRGN, by bacterial species

**Table 4:**
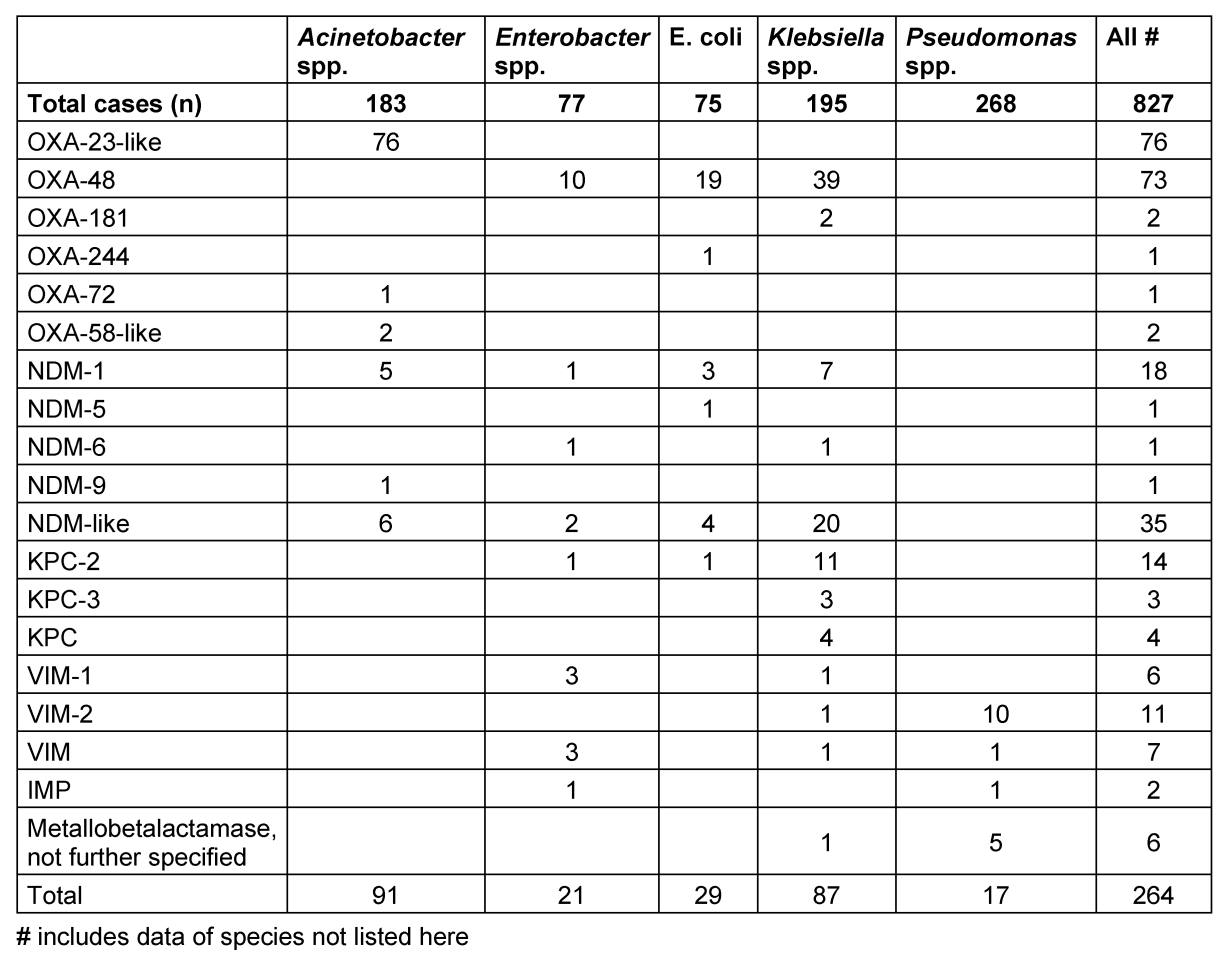
Detection of carbapenemases for different reported bacterial species
